# Enhancing psychological health and cognitive inhibition in college students: insights from mindfulness training and high-intensity interval training

**DOI:** 10.3389/fpsyg.2025.1528049

**Published:** 2025-05-02

**Authors:** Yintu Bao, Jianqian Sun, Xiaochuan Zhang

**Affiliations:** ^1^Gymnasium of Physical Education Teaching Department, Dalian Minzu University, Dalian, China; ^2^Department of Aviation Physical Education, Aviation University of Air Force, Changchun, China; ^3^Teaching Evaluation Center, Aviation University of Air Force, Changchun, China

**Keywords:** psychological health, cognitive inhibition, high-intensity interval training, mindfulness training, college students

## Abstract

**Background:**

This study aimed to examine the effects of mindfulness training (MT) and high-intensity interval training (HIIT) on enhancing psychological health and cognitive inhibition in college students.

**Methods:**

A total of 132 undergraduates were recruited and randomly assigned to three groups: MT group, HIIT group, and control group. Both the MT and HIIT groups received training twice a week for 6 weeks, with each session lasting 1 h. The control group did not undergo MT or HIIT training during the experimental period. The Beck Depression Inventory (BDI-II), Beck Anxiety Inventory (BAI), Stop Signal tasks, and Flanker tasks were assessed at baseline, at 6 weeks (post-test), and 6 weeks after the end of the intervention (follow-up).

**Results:**

Both MT and HIIT were effective in alleviating depression and anxiety in college students, with MT showing a significant improvement in psychological health after intervention. Both MT and HIIT effectively improved the response inhibition accuracy of college students, with similar effects. However, neither MT nor HIIT affected the response inhibition reaction time. MT was more effective than HIIT in improving interference inhibition accuracy, but neither MT nor HIIT had an effect on the interference inhibition reaction time.

**Conclusion:**

Within-group analyses demonstrated significant pre-post reductions in depression and anxiety scores following both MT and HIIT interventions. While between-group comparisons suggested a trend toward greater symptom improvement in the MT group at post-test, the differences did not reach statistical significance at follow-up, possibly due to the limited sample size and intervention duration. These preliminary findings warrant replication in larger-scale trials with extended observation periods.

## 1 Introduction

In recent years, there has been an increasing focus on the psychological health of college students, particularly concerning the prevalence of depression and anxiety. Psychological health is defined as a condition in which all aspects of psychological wellbeing and functioning are in an optimal or normative state (Handler and Potash, 1999). In this study, research on college students’ psychological health specifically refers to the relief of anxiety and depression within this population. Numerous studies and surveys indicate a worrying trend: an escalating number of students reporting symptoms associated with this psychological health. The pressures of college life, combined with societal changes, contribute significantly to this phenomenon. The increased autonomy and responsibility that come with college life can amplify feelings of uncertainty and anxiety, particularly among younger students who may be experiencing such independence for the first time. Academic pressures are among the most cited causes of student stress. Numerous studies indicate a concerning trend: an increasing number of students reporting symptoms related to psychological health (Liu and Wang, 2024; Zhang and Hu, 2024). The pressures of college life, combined with societal changes, contribute significantly to this issue (Xu et al., 2024). The heightened autonomy and responsibility associated with college can amplify feelings of uncertainty and anxiety, particularly among younger students experiencing such independence for the first time. Academic pressures are frequently cited as the primary causes of student stress (van Tienoven et al., 2024). The pressure to succeed can foster a pervasive fear of failure, manifesting as anxiety or depression (Zhao et al., 2024). Moreover, competitive academic environments can heighten feelings of inadequacy, leading students to question their abilities (Luna Silveira et al., 2022). Depression and anxiety among college students arise from a complex interplay of academic, social, and personal factors (Kakemam et al., 2024; Li and Sun, 2023). Addressing these issues requires a comprehensive approach, including social support, physical exercise, and accessible counseling services (Carek et al., 2011; Iqbal et al., 2015; Liu et al., 2024). Implementing such strategies can create a more supportive college environment that enhances both academic achievement and personal wellbeing.

High-intensity interval training (HIIT) has gained prominence in recent years as not only an efficient physical exercise regimen but also as a potent tool for psychological health improvement among various demographics, including college students (Leahy et al., 2020). The period of college is often associated with heightened stress and psychological health challenges, such as anxiety and depression, which can impair both academic performance and quality of life (Barbosa-Camacho et al., 2022). Emerging research underscores the potential of HIIT as a beneficial intervention for these issues, highlighting its positive effects on reducing symptoms of anxiety and depression (Andreato and Esteves, 2024). HIIT involves short bursts of intense exercise followed by rest or low-intensity periods, making it an appealing option for busy college students who often struggle with time constraints. Studies indicate that the physiological benefits of HIIT, such as enhanced cardiovascular fitness and hormonal regulation, may play a crucial role in its psychological health benefits (Manferdelli et al., 2020). The intense physical activity can lead to increased endorphin levels and neurogenesis, thereby improving mood and decreasing anxiety (Leal-Galicia et al., 2019). Moreover, HIIT has been shown to reduce cortisol levels, a stress hormone often elevated during periods of anxiety and depression (Bonato et al., 2020; Oniz et al., 2024). In college students, this could translate to better stress management and resilience. While more longitudinal studies are needed to fully understand the extent of HIIT’s benefits, current evidence suggests that incorporating HIIT into the lifestyle of college students could serve as a holistic approach to mitigating anxiety and depression (Borrega-Mouquinho et al., 2021). As an accessible, time-efficient, and effective exercise model, HIIT offers a promising avenue for supporting mental wellbeing and cognitive health in the college population. Nevertheless, personalized interventions, considering individual fitness levels and psychological health conditions, will be essential to maximize these benefits.

Mindfulness training (MT) has emerged as a promising intervention for reducing anxiety and depression among college students, a demographic particularly vulnerable to psychological health issues due to academic pressures, social dynamics, and significant life transitions. Research consistently demonstrates that MT, which emphasizes present-moment awareness and acceptance, effectively reduces symptoms of anxiety and depression (Caliskan et al., 2024). The origins of MT can be traced back to ancient Eastern philosophical and religious traditions and have been scientifically adapted in modern times as significant interventions in psychology and medicine. MT focuses on the premise that stress can be alleviated by observing physical sensations, emotional changes, mental activity, and the nature of phenomena (Cohen and Miller, 2009). Mindfulness-based stress reduction (MBSR) was developed in the late 1970s by American scientist Dr. Jon Kabat-Zinn. He secularized the Buddhist practice of mindfulness meditation and integrated it with modern psychology, medicine, and neuroscience to create a structured curriculum aimed at helping patients manage stress, pain, and illness through scientifically based training (Trowbridge et al., 2017). Mindfulness-based cognitive therapy (MBCT) originated in the 1990s when psychologists Mark Williams, John Teasdale, and Zindel Segal combined mindfulness with cognitive-behavioral therapy, following their study of Kabat-Zinn’s MBSR program, to prevent depression recurrence (Teasdale et al., 2000). MBSR and MBCT are among the most studied programs, showing significant reductions in anxiety and depressive symptoms in students (Maloney et al., 2024; Tao et al., 2022). Studies suggest that MT fosters neuroplasticity, leading to changes in brain regions involved in attention, self-awareness, and emotional processing. This neurobiological basis underscores the potential for durable improvements in psychological health (Yu et al., 2021). In addition, MT enhances resilience, enabling students to better manage stress and reduce the likelihood of anxiety and depression exacerbation. A meta-analysis of multiple studies supports these findings, showing moderate-to-large effect sizes for MT in reducing anxiety and depression symptoms in student populations (Johannsen et al., 2022; Liu et al., 2023a,b). Importantly, MT is adaptable and cost-effective, making it accessible to diverse student populations. Institutions are increasingly incorporating MT programs into their wellness offerings, recognizing their role in fostering not just psychological health but also academic performance and overall wellbeing. While there is compelling evidence supporting the efficacy of MT, further research is needed to optimize program delivery, explore long-term outcomes, and identify which students benefit most. In addition, integrating MT into broader psychological health strategies ensures a comprehensive approach to addressing the complex spectrum of student psychological health needs. MT improves cognitive inhibition by enhancing attentional control and emotion regulation while also reducing thought rumination (Chiesa and Serretti, 2010). In contrast, HIIT influences cognitive inhibition by increasing brain-derived neurotrophic factor (BDNF) levels through physical exercise (Li et al., 2022). Although MT and HIIT have distinct mechanisms, they both target different aspects of cognitive inhibition. Comparing these interventions can elucidate their effects on different intervention pathways. Consequently, this study examines the similarities and differences between MT and HIIT in relation to college students’ mental health and cognitive inhibition through a comparative analysis.

Inhibition is a key component of cognitive function that involves self-control and the ability to regulate thoughts and actions (Chu et al., 2015). Inhibition is crucial in regulating emotional responses and facilitating adaptive functioning (Mata-Marin et al., 2023). In individuals with depression, there often exists a heightened negative bias and rumination, leading to impaired cognitive inhibition where the individual finds it challenging to suppress negative thoughts (Johansson et al., 2023). Research shows that impaired inhibitory control may exacerbate both anxiety and depression, possibly creating a feedback loop where decreased inhibition leads to increased symptoms, which in turn further diminishes inhibitory capacity (Overstreet et al., 2023; Sun et al., 2023). Improved inhibition can help students manage academic workload pressures and social challenges more effectively. Preliminary studies suggest that the enhanced blood flow and neuroplasticity associated with regular HIIT sessions may contribute to better executive function, including improved inhibitory control (Guo et al., 2023). Research has also shown that MT can also be effective in improving an individual’s executive function (Gallant, 2016).

HIIT and MT are two interventions that have gained attention for their potential benefits in reducing anxiety and depression. HIIT involves short bursts of intense exercise followed by periods of rest or low-intensity activity. This form of exercise has been shown to improve psychological health by increasing endorphin levels, enhancing mood, and reducing stress, which can alleviate symptoms of anxiety and depression (Luo et al., 2019). Its time-efficient nature makes it appealing to college students seeking quick stress-relief strategies. MT focuses on cultivating positive thoughts, gratitude, and present-moment awareness. It has been linked to decreased rumination and negative thinking, which are significant contributors to anxiety and depression. Studies suggest that MT can help students develop healthier coping mechanisms, foster emotional regulation, and improve overall psychological wellbeing (Erguler et al., 2023). However, which of the two interventions—MT or HIIT—has a more effective impact on the psychological health and cognitive inhibition of college students remains unclear. The aim of this study is to examine the impact of MT and HIIT on psychological health and cognitive inhibition in college students. Specifically, the study assessed the effects of MT and HIIT on psychological health and cognitive inhibition in college students. The outcome of our study was to evaluate the effects of MT and HIIT on depression, anxiety, and cognitive inhibition in college students from baseline to the follow-up period. We hypothesize that MT and HIIT can effectively alleviate psychological health issues and improve cognitive inhibition in college students.

## 2 Methods

### 2.1 Research design

This study adopted a randomized controlled experimental design, which included an MT group, HIIT group, and control group. The students were divided into three groups by a random method. The randomization was grouped according to an online resource.^1^ This study was conducted using a single-blind method. In addition, a single-blind method was employed during the study to minimize bias, and only the organizers of the experiment, including the physical education teacher and the psychology teacher responsible for the MT, were aware of the existence of the HIIT, MT, and control groups. The college students were unaware of the other groups’ existence. Depression, anxiety, and cognitive inhibition measures were administered in three separate sessions: baseline, post-test (6 weeks), and follow-up (6 weeks after the end of intervention).

### 2.2 Participants

**Figure 1:**
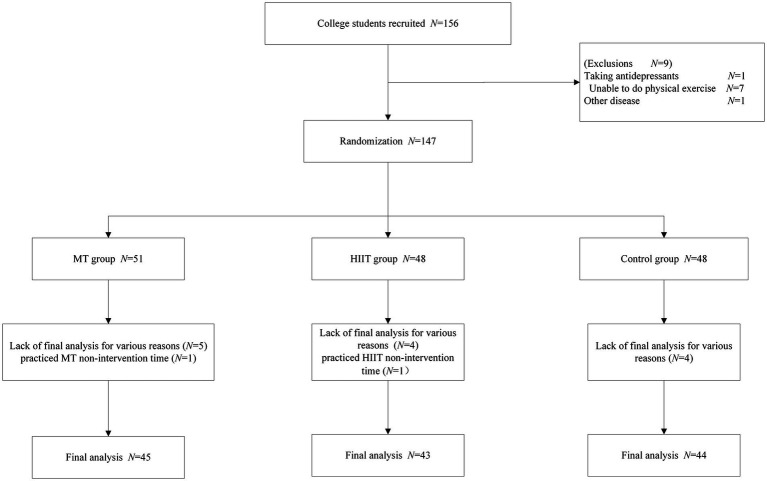
Participant flowchart across the study.

*A priori* power analysis (G*Power3.1.9.7) was used to calculate the study’s required sample size. The chosen parameters were as follows: (1) effect size *f* = 0.25;(2) *α* error probability of 0.05; (3) power of 0.85; (4) number of groups of 3; and (5) number of measurements of 3. After calculation, a sample size of 110 can produce statistical significance. Considering that college students may withdraw from the experiment for some reasons while participating in the experiment, the sample size needs to be appropriately expanded when recruiting. It was estimated that the attrition rate of college students was approximately 20%, and after calculation, at least 132 college students who could accept the experimental test were needed. Participants were recruited for this study starting in March 2023. College students at the College Student Psychological Health Center were recruited. All participants underwent assessment based on the Diagnostic and Statistical Manual of Mental Disorders (DSM-V), and all participants in the experiment were diagnosed with mild depressive symptoms. Among recruited college students, those who met the following criteria would be excluded: (1) taking pharmacological treatment (all participants taking anti-anxiety and anti-depressant pharmacological will be excluded); (2) being unable to do physical exercise with physical defects (such as fractures and heart disease); (3) practicing HIIT more than twice weekly; (4) having received MT treatment; and (5) suffering from other mental diseases. A total of 156 subjects were recruited at the beginning. According to the screening conditions, finally, 132 college students were qualified for the analysis, including 45 in the MT group, 43 in the HIIT group, and 44 in the control group. Several participants dropped out of each group. The primary reason was their inability to persist with this action, resulting in their automatic removal from the experiment. Six participants from the MT group (five were absent during the final test and one practiced MT non-intervention time), five from the HIIT group (four were absent during the final test and one practiced HIIT non-intervention time), and four participants from the control group (all absent during the final test) will be excluded from the final analysis. The participant flowchart across the study is shown in Figure 1.

### 2.3 Study methodology

Studies have demonstrated that engaging in 2 min of high-intensity interval training (HIIT) per week over a 6-week period can reduce anxiety sensitivity in adults with asthma (O'Neill and Dogra, 2020). In our study, we developed an intensified training regimen comprising a 25 min session, which includes 15 min of HIIT. To ensure consistency between the mindfulness training (MT) and HIIT durations, we set the MT session length to 25 min as well.

#### 2.3.1 The design plan of HIIT

The intervention plan for MT was negotiated by a physical education teacher. For example, first, warm-up (5 min): Start on the treadmill at an easy pace and gradually increase the speed until the subject’s heart rate reaches 50–60% of the subject’s maximum heart rate. Then maintain this speed for a 5 min warm-up. Second, HIIT (15 min): run at full speed for 30 s, as fast as possible, then reduce the speed to a slow walk or rest for 30 s. Repeat the process, 5 training process for a group, a total of 3 sets of training. During the sprint, try to keep your speed consistent and do not decrease it too much. During the rest or slow walk, try to keep your body moving and do not stop in place. Third, cool-down phase (5 min): reduce the speed and incline of the treadmill, so that the heart rate is gradually reduced until it returns to the resting state. The 12 HIIT sessions were done on a treadmill to ensure that the HIIT was of a certain intensity, so we were only able to change the intervals, and the related workouts are presented in Table 1. Since the warm-up and cool-down phases were consistent for each session, we do not show the warm-up and cool-down phases in Table 1.

**Table 1:** 12 sessions of HIIT and MT.

Sessions	HIIT	MT
1	HIIT: High-intensity running: 30 s Low-intensity intervals: 30 s Repetitions: 15 sets	Goal: Establish initial concentration through breathing. Method: Natural breath awareness, focusing on the sense of airflow from nostril breathing, bringing it back gently when distracted.
2	HIIT: High-intensity running: 30 s Low-intensity intervals: 45 s Repetitions: 12 sets	Goal: To connect the body with consciousness. Method: Scan progressively from toes to top of head, focusing on the feeling of tightness/relaxation in each area in turn, without judgment.
3	HIIT: High-intensity running: 40 s Low-intensity intervals: 50 s Repetitions: 10 sets	Goal: Expanding present-moment awareness through listening. Method: Open awareness of environmental sounds, distinguishing between near and far, high and low tones, without getting caught up in thinking.
4	HIIT: High-intensity running: 20 s Low-intensity intervals: 40 s Repetitions: 15 sets	Goal: Observe emotions without getting involved. Method: Think back to a recent event, recognize an emotion rising (e.g., irritation/joy), meditate on “this is what it feels like to be…,” imagine the emotion passing through as a cloud, and return to your breath.
5	HIIT: High-intensity running: 45 s Low-intensity intervals: 30 s Repetitions: 12 sets	Goal: Integrate awareness into dynamic movement. Method: Focus on the muscle changes of lifting, moving, and landing the foot.
6	HIIT: High-intensity running: 25 s Low-intensity intervals: 35 s Repetitions: 15 sets	Goal: To create a connection between self and others. Method: Extend blessings to family, strangers and even “difficult people.”
7	HIIT: High-intensity running: 35 s Low-intensity intervals: 55 s Repetitions: 10 sets	Goal: Strengthening concentration resilience. Method: Deliberately create distractions (e.g., playing white noise), focus on your breathing, observe how distractions arise, and practice “pull-back” movements.
8	HIIT: High-intensity running: 15 s Low-intensity intervals: 30 s Repetitions: 20 sets	Goal: Global view beyond a single anchor point. Method: Meditate while being aware of breath, body, and sound, without focusing on a single point, and record the strongest feelings in your consciousness.
9	HIIT: High-intensity running: 50 s Low-intensity intervals: 40 s Repetitions: 10 sets	Goal: Containerize difficult emotions. Method: Imagine negative emotions in a “transparent container” and observe their color and shape.
10	HIIT: High-intensity running: 30 s Low-intensity intervals: 60 s Repetitions: 10 sets	Goal: Expansion of time perception. Method: Estimate 1 min duration with eyes closed and compare to actual time. Perceive the difference between subjective and objective time.
11	HIIT: High-intensity running: 20 s Low-intensity intervals: 25 s Repetitions: 20 sets	Goal: Dynamic Positive Mind Integration. Method: Walk slowly while counting your breath (1 count when your foot touches the ground), keeping the movement synchronized with the counting. Play soft music while being aware of the interaction between the rhythm of the breath and the melody.
12	HIIT: High-intensity running: 35 s Low-intensity intervals: 55 s Repetitions: 10 sets	Goal: Integration review. Method: free choice of the most effective combination of the 3 methods practiced in the first 11 times.

#### 2.3.2 The design plan of MT

The intervention plan for MT was negotiated by a psychology teacher. The psychology teacher holds a professional certification in MT. The relevant MT courses are listed below: For example, first, preparation phase (5 min): Sit or lie down and allow the subject’s body to be comfortable and relaxed. Close your eyes and take a few deep breaths to allow the subject’s mind to begin to calm down. Second, Meditation phase (15 min): Overall perception (3 min): become aware of the rise and fall of the chest/abdomen during breathing; Refinement of focus (10 min): focus on the tactile sensation of airflow at the edge of the nostrils (cold/warm, flow rate, contact points). Third, closing phase (5 min): Gradual relaxation of the body: At the end of the meditation, gradually relax all parts of the body, starting with the head and working your way down to the toes. Thank yourself: While relaxing your body, thank yourself for your efforts and patience, and realize that meditation is a valuable opportunity for self-care and growth. Slowly open your eyes: Finally, slowly open your eyes and gradually adjust to your surroundings. The 12 MT sessions were all done in a quiet gymnasium, and the related MT is presented in Table 1. Since the preparation phase and closing phase were consistent for each session, we do not present the preparation phase and closing phase in Table 1.

Both the MT group and the HIIT group were trained in a quiet gymnasium, and the two groups were in the same environment. The control group did not undergo any intervention of HIIT and MT. After all the tests, the control group then performed 12 classes of HIIT or MT for 6 weeks. Participants were asked to sign a paper version of informed consent to ensure that their participation in the study was voluntary and to protect their rights as subjects. Information obtained from the participants was treated with strict confidentiality and solely used for research purposes. Moreover, it was explicitly stated that no commercial use would be made out of the information provided. Participants were informed about their right to withdraw from the study in case of any discomfort. The study has been approved by the Ethics Committee of Dalian Minzu University, and the study procedure was in accordance with the Ethical Standards of the Institutional and National Research Committee and the 1964 Helsinki Declaration and its later amendments or comparable ethical standards.

### 2.4 Measuring tools

The Beck Depression Inventory (BDI-II) was used to measure the depression level of college students, which had been validated in a college student population (O'Hara et al., 1998). BDI-II includes 21 items, which were completed by the subjects themselves. Each item was scored on a scale of 0–3. Depression classification criteria: 0–13 points meant no depression, 14–19 points meant mild depression, 20–28 points meant moderate depression, and 29–63 points meant severe depression. The higher the score, the greater the symptoms of depression.

The Beck Anxiety Inventory (BAI) was used to measure the anxiety level of college students. The BAI has been widely used to measure people’s anxiety (Beck et al., 2001). BAI can be divided into 21 items in total, and each item was between 0 and 4 points. The higher the score is, the more severe the anxiety is. Total scores: nothing/mild (0–15), moderate (16–25), and severe (26–63). The study showed that BAI had high reliability and validity and can be well applied to individual anxiety measurement in the population.

Response inhibition was assessed through the Stop Signal Task (Cabel et al., 2000). During the test procedure, the symbols “>“and “<“appeared in the center of the screen. Participants were instructed to press the “A” key when “>“appeared and the “L” key when “<“appeared. Each symbol appeared for 2000 ms, and if no response was made within that time, it was recorded as an error. If a symbol appeared in the center of the screen and the stop signal “di” was heard, participants were instructed not to make any response. The primary measurements include the accuracy of keystroke responses and the reaction time.

Interference inhibition was assessed through the Flanker tasks (Eriksen and Eriksen, 1974). Participants were presented with seven symbols in the center of the screen. They were instructed to press the A key when consistent symbols appeared (“>>>>>>> > or <<<<<<<“) and the L key when inconsistent symbols appeared (“>> > <>> > or <<<> < <<“). The experimental measurements included the accuracy of key press responses and the reaction time associated with accuracy.

### 2.5 Statistical analysis

Statistical analysis of the data was conducted using SPSS 26.0. The baseline levels of the MT group, HIIT group, and control group were analyzed by one-way analysis of variance (ANOVA). The Shapiro–Wilk test was used for the standard normal distribution of the data, and Levene’s test was used for the homomorphic distribution. Within-subject effect tests were calibrated using Greenhouse–Geisser adjustments. Changes in anxiety, depression, and inhibition after intervention were compared among the three groups by repeated measures analysis of variance. Mauchly was used to perform the sphericity test, and if the sphericity test was not met, the Greenhouse–Geisser was used for analysis. In terms of significance, Bonferroni *post-hoc* analysis was used. Significant levels were expressed as *p*-values. Eta squared (𝜂^2^) calculates the effect sizes of significant main effects and interactions. According to Correll, an 𝜂2 value greater than 0.01 indicates a small effect, greater than 0.06 indicates a medium effect, and greater than 0.14 indicates a large effect (Correll et al., 2022). We need to finally observe the effects of HIIT and MT on individuals, and we focus on comparative studies, so we use per-protocol analysis.

## 3 Results

### 3.1 Participant characteristics

There were no significant differences in age (*F* (2,129) = 0.875, *p* = 0.412), gender (χ^2^ (2) = 0.438, *p* = 0.803), body height (F (2,129) = 1.312, *p* > 0.108), body mass (F (2,129) = 1.113, *p* = 0.235), BMI (F (2,129) = 1.213, *p* = 0.209), vital capacity (F (2,129) = 0.516, *p* = 0.659), resting heart rate (F (2,129) = 1.012, *p* = 0.376), and physical activity (F (2,129) = 0.976, *p* > 0.543). The relevant results are shown in Table 2.

**Table 2:** Baseline characteristics of subjects (M ± SD).

Characteristics	MT group (*N* = 45)	HIIT group (*N* = 43)	Control group (*N* = 44)	*p*-value
Age (years)	21.3 *±* 1.23	20.53 *±* 1.86	21.32 *±* 1.77	0.412
Gender (males/females)	23/22	25/18	24/20	0.513
Body height (cm)	173.1 *±* 3.10	171.5 *±* 3.23	172.7 *±* 3.11	0.108
Body mass (kg)	63.8 *±* 2.74	62.3 *±* 2.83	64.3 *±* 3.49	0.235
BMI (kg/m^2^)	21.6 *±* 1.14	21.3 *±* 1.06	21.7 *±* 1.97	0.209
Vital capacity (ml)	3816.9 *±* 24.67	3899.4 *±* 25.37	3817.29 *±* 24.12	0.659
Resting heart rate	75.5 *±* 3.13	74.6 *±* 3.46	74.7 *±* 3.25	0.376
Physical activity (avg. min/day)	40.7 *±* 2.16	46.6 *±* 3.74	45.9 *±* 3.37	0.543

M, mean; SD, standard deviations; *N*, number; BMI, body mass index.

### 3.2 Outcomes

#### 3.2.1 BAI scores

**Figure 2:**
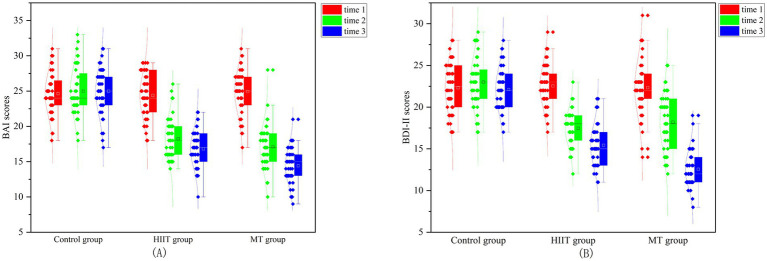
BAI and BDI-II scores in college students among three groups. **(A)** The BAI scores; **(B)** The BDI-II scores.

A repeated-measures ANOVA on the BAI scores revealed a significant main effect of measurement time (*F* (2,128) = 157.235, *p* < 0.001, 𝜂^2^ = 0.711). Scores at the follow-up (18.15 ± 3.13) and post-test (20.12 ± 3.03) were lower than those at the baseline measurement (25.15 ± 3.43). The interaction between group and measurement time was also significant (*F* (4, 258) = 27.785, *p* < 0.001, η^2^ = 0.301). *Post-hoc* tests within groups showed that follow-up scores (14.20 ± 3.05) in the MT group were lower than post-test scores (17.37 ± 3.16) and were significantly different (*p* = 0.021). Both follow-up and post-test scores were significantly lower than baseline scores (25.30 ± 3.76), with all comparisons showing significant differences (*p* < 0.05). In the HIIT group, follow-up (16.80 ± 3.18) and post-test scores (17.42 ± 2.99) were not significantly different from one another (*p* = 0.253) but were both significantly lower than baseline scores (24.90 ± 4.03) (*p* < 0.05). A comparison between groups indicated no significant difference in post-test scores (*p* = 0.415) between the MT and HIIT groups. However, there was a significant difference in follow-up scores (*p* = 0.017). This demonstrates that the MT intervention can effectively and consistently alleviate self-assessment anxiety symptoms in college students. The BAI scores for the three groups are depicted in Figure 2A.

#### 3.2.2 BDI-II scores

A repeated-measures ANOVA on the BDI-II scores revealed a significant main effect of measurement time (*F* (2,128) = 155.030, *p* < 0.001, 𝜂^2^ = 0.708). Scores at follow-up (17.84 ± 3.59) and post-test (20.19 ± 3.45) were lower than those at baseline measurement (25.32 ± 3.11). The interaction between group and measurement time was significant (*F* (4,258) = 28.946, *p* < 0.001, 𝜂^2^ = 0.311). A *post-hoc* comparison within groups revealed that, for the MT group, follow-up scores (13.33 ± 2.98) were significantly lower than post-test scores (17.32 ± 2.84), with *p* = 0.002. Both follow-up and post-test scores were significantly lower than baseline scores (22.71 ± 3.23), all with *p* < 0.05. For the HIIT group, follow-up (17.81 ± 3.26) and post-test (18.26 ± 2.83) scores were not significantly different from each other, but both were significantly lower than baseline scores (23.55 ± 2.99), with all *p* < 0.05. Between-group comparisons revealed no significant difference in post-test scores between the MT and HIIT groups (*p* = 0.109), but there was a significant difference in follow-up scores (*p* = 0.002). These findings suggest that MT intervention can effectively and consistently alleviate self-rated depression symptoms in college students. The BDI-II scores for the three groups are shown in Figure 2B.

#### 3.2.3 Stop signal task accuracy and reaction time

**Figure 3:**
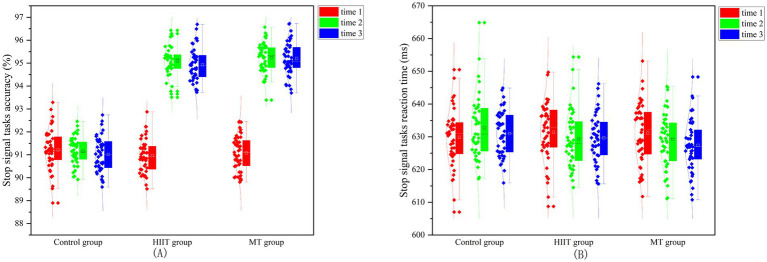
Accuracy and reaction time of the signal stop tasks in college students among three groups. **(A)** The stop signal task accuracy; **(B)** The stop signal task reaction time.

A repeated-measures ANOVA on the accuracy of the stop signal tasks revealed a significant main effect of measurement time (*F* (2,128) = 515.108, *p* < 0.001, 𝜂^2^ = 0.889). Accuracy at follow-up (93.25 ± 0.32) and post-test (93.11 ± 0.23) was higher than at baseline (91.23 ± 0.52). The interaction between group and measurement time was significant (*F* (4, 258) = 44.607, *p* < 0.001, η^2^ = 0.409). A *post-hoc* analysis within groups found that, for the MT group, follow-up (95.13 ± 0.54) and post-test (95.27 ± 0.42) accuracy did not differ significantly from each other (*p* = 0.413), but both were significantly higher than baseline accuracy (91.23 ± 0.87), with *p* < 0.05. Similarly, for the HIIT group, follow-up (95.19 ± 0.47) and post-test (95.29 ± 0.59) accuracy did not differ significantly (*p* = 0.409), but both were significantly higher than baseline accuracy (90.16 ± 0.63), with *p* < 0.05. Comparisons between the groups showed no significant difference in post-test and follow-up accuracy between the MT and HIIT groups (*p* > 0.05). This suggests that MT and HIIT were equally effective in improving response inhibition accuracy. For the repeated-measures ANOVA on reaction time for the stop signal tasks, the main effect of measurement time was not significant (*F* (2, 128) = 1.139, *p* = 0.323, η^2^ = 0.017). The interaction between group and measurement time was also not significant (F (4, 258) = 1.558, *p* = 0.186, η^2^ = 0.024). This indicates that neither MT nor HIIT training sufficiently reduced response inhibition reaction time. The stop signal tasks’ accuracy and reaction time for the three groups are shown in Figures 3A,B.

#### 3.2.4 Flanker task accuracy and reaction time

**Figure 4:**
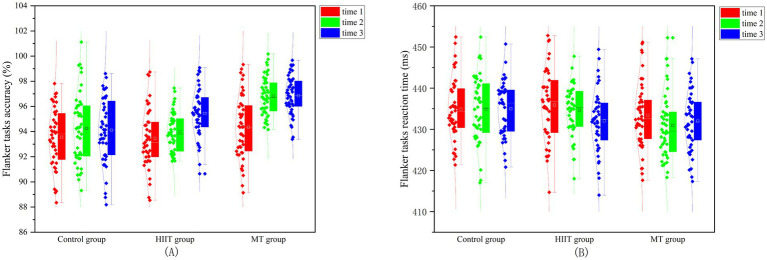
Accuracy and reaction time of the Flanker tasks in college students among three distinct groups. **(A)** The Flanker task accuracy; **(B)** The Flanker task reaction time.

A repeated-measures ANOVA on the accuracy of the Flanker tasks revealed a significant main effect of measurement time (*F* (2,128) = 19.884, *p* < 0.001, 𝜂^2^ = 0.237). The accuracy at measurement follow-up (95.32 ± 1.21) and post-test (95.87 ± 1.23) was higher than at measurement baseline (94.14 ± 1.19). A significant interaction between group and measurement time was observed (*F* (4,258) = 4.418, *p* = 0.002, η^2^ = 0.064). *Post-hoc* tests revealed that, within the MT group, both the follow-up (97.13 ± 1.09) and post-test (97.27 ± 1.12) accuracies were not significantly different (*p* = 0.104). However, both were significantly higher than the baseline accuracy (94.23 ± 1.32), with a significance level of *p* < 0.05. For the HIIT group, follow-up (95.10 ± 1.99), post-test (95.13 ± 1.21), and baseline (94.35 ± 1.56) accuracies were not significant (*p* > 0.05). Comparison between groups revealed a significant difference between the MT group and HIIT group in both post-test (*p* < 0.05) and follow-up (*p* < 0.05) accuracies. It was shown that MT was better than HIIT in improving the effect of interference inhibition in accuracy. For the repeated-measures ANOVA on reaction time for the Flanker tasks, the measurement time main effect was not significant (F (2,128) = 1.864, *p* = 0.159, 𝜂^2^ = 0.028). The interaction between group and measurement time was not significant (F (4,258) = 1.196, *p* = 0.313, 𝜂^2^ = 0.018). It was shown that MT and HIIT are not sufficient to cause a decrease in interference inhibition reaction time. The Flanker task accuracy and reaction time of the three groups are shown in Figures 4A,B.

## 4 Discussion

The study examined the impact of a 6-week MT and HIIT intervention on psychological health and cognitive inhibition in college students. Follow-up measurements were conducted 6 weeks after the intervention concluded. Both the MT and HIIT groups demonstrated significant reductions in anxiety and depression symptoms post-intervention. Specifically, both groups showed significant improvements in depression symptoms with no substantial differences between them initially. However, at the follow-up, while the MT group continued to show improvements, the HIIT group did not. This resulted in a significant difference in depression scores, favoring the MT group. These results highlight the effectiveness of MT in reducing depression symptoms in college students and suggest continued benefits even after the intervention ends.

Previous studies have consistently shown the effectiveness of MT in reducing symptoms of anxiety, depression, and related conditions (Guney et al., 2022; Yildirim and Yildiz, 2022). A meta-analysis indicated that the effects of MT on social depression disorder can be maintained for up to 12 months, providing evidence for the long-term efficacy of MT (Liu et al., 2021). Rawtaer conducted an intervention study with the elderly using MT to assess their levels of anxiety and depression. The study revealed that MT had a pronounced effect on improving anxiety and depression symptoms in the elderly population (Rawtaer et al., 2015). When college students ceased practicing MT, they also showed a significant decrease in anxiety symptoms. Shapira discovered that MT has the potential to alleviate anxiety and stress in veterans diagnosed with depression (Shapira et al., 2022). Consequently, MT can be utilized as a supplementary approach in the treatment of depression. According to Koszycki’s research, MT was found to be more effective than CBT in reducing anxiety levels in individuals (Koszycki et al., 2021). Hence, MT can partially substitute CBT in the treatment of anxiety in individuals. Why does the MT intervention continue to alleviate depression and anxiety even after the practice is discontinued? In a 4-year follow-up study, Solhaug et al. reported that immediate cessation of MT had a sustained effect on reducing mental stress in students (Solhaug et al., 2019). In the current study, one possible explanation is that MT has a long-term impact on individuals, leading to sustained improvement in their depression and anxiety symptoms post-practice cessation.

In the response inhibition test, no significant difference was observed in the accuracy of response inhibition between the MT group and the HIIT group during both the post-test and follow-up assessments. Regarding the reaction time of response inhibition, neither MT nor HIIT has an impact on it. The observed phenomenon may be attributed to the baseline design of response inhibition, which was set at a high level, leading to ceiling effects. In psychology, ceiling effects occur when test questions are too simple, causing most participants to achieve high scores and thus failing to effectively distinguish individual differences (Hammers et al., 2024). This effect diminishes a test’s discriminatory power, differentiation, and validity. Consequently, it is possible that both MT and HIIT did not exhibit differences in reaction time related to response inhibition due to this ceiling effect. In terms of interference inhibition, MT demonstrates a significant improvement in the accuracy of interference inhibition, whereas HIIT does not show any improvement in this aspect. Regarding the reaction time of interference inhibition, neither MT nor HIIT had any impact on it, as observed in this study. Currently, there is a greater emphasis in studies on the impact of HIIT on executive function rather than cognitive inhibition. Research has demonstrated a significant ameliorative effect of HIIT on executive function (Hsieh et al., 2021; Wang et al., 2023). Regarding research on cognitive inhibition through MT, Zheng discovered that short-term MT enhanced subjects’ response inhibition using the event-related potential technique (Zheng et al., 2023). Conversely, Boeckler’s study revealed that MT was effective in improving individuals’ working memory, but it did not have an impact on response inhibition (Boeckler and Singer, 2022). Our study indicates that MT has a certain degree of influence on cognitive inhibition. This could be due to the activation of the frontal area of the brain, which is associated with cognitive inhibition. Consequently, practicing MT can effectively enhance cognitive inhibition (Mehnert et al., 2013; Semmel et al., 2022). The study’s conclusion supports the hypothesis that MT can effectively enhance psychological health and improve cognitive inhibition. Following a 6-week intervention, both the MT and HIIT groups experienced significant reductions in symptoms of anxiety and depression, demonstrating notable improvements compared to their baseline levels. This finding suggests that both MT and HIIT can effectively alleviate symptoms of depression and anxiety disturbances in college students. However, even after the practice was discontinued, the MT group’s anxiety and depression scores continued to decrease. This difference may be attributed to the persistent psychological effects of MT (Hong et al., 2023; Lothes et al., 2023; Watkins et al., 2022). Consequently, the benefits of MT endured beyond its discontinuation, providing sustained relief from symptoms of anxiety and depression in this group.

MT can be regarded as a form of psychotherapy. In the MT group, college students used mindfulness techniques to alleviate anxiety and depressive symptoms effectively due to their proficiency with the intervention. Both MT and HIIT enhanced response inhibition accuracy, although only MT improved interference inhibition accuracy. This finding suggests that MT can effectively enhance college students’ resistance to interference (Greenberg et al., 2019; Nigol and Di Benedetto, 2020). Regarding cognitive inhibition reaction time, the study found that neither response nor interference inhibition, in both MT and HIIT, improved the participants’ reaction time. This suggests that neither MT nor HIIT can enhance reaction time, possibly due to the short training duration being insufficient for significant improvement. However, the study has limitations. First, there was limited discussion about the target demographic of the MT intervention, particularly its effectiveness for the elderly. Second, the reliance on self-reported questionnaires for anxiety and depression may introduce some bias to the results. Third, our statistical approach relied on per-protocol analysis and did not perform an intention-to-treat (ITT) analysis, which may have overestimated the treatment effect. This study aimed to conduct a preliminary investigation into the effects of MT and HIIT on the psychological health and cognitive inhibition of college students. The study demonstrated that MT and HIIT can effectively enhance the psychological health and cognitive inhibition of college students.

## 5 Conclusion

Following a 6-week intervention, both the MT and HIIT groups showed improvements in depression and anxiety. However, 6 weeks after the intervention concluded, the MT group maintained these improvements, whereas the HIIT group did not. In terms of cognitive inhibition, both MT and HIIT effectively enhanced response inhibition accuracy. Regarding interference inhibition, MT improved the accuracy of college students’ interference inhibition, whereas HIIT did not show any improvement in this area. These findings indicate that MT has a more substantial positive effect on enhancing the psychological health and cognitive inhibition of college students.

## Data Availability

The raw data supporting the conclusions of this article will be made available by the authors, without undue reservation.

## Footnotes

^1^ http://www.randomization.com
